# Diaphragm Dysfunction After Cardiac Surgery: Revisiting an Underrecognized Complication

**DOI:** 10.21470/1678-9741-2025-0227

**Published:** 2025-12-10

**Authors:** Khaled Alebrahim

**Affiliations:** 1 King Abdulaziz University, Jeddah, Saudi Arabia. E-mail: dr.k.ebrahim@gmail.com

Dear Editor,

We read with great interest the article by Somers et al.^[1]^, *“Diaphragm Dysfunction After Cardiac Surgery”*, which presents important clinical observations regarding the incidence and spontaneous resolution of postoperative diaphragmatic elevation. Their finding that 27% of patients developed new diaphragm elevation, 65% of whom recovered without intervention, emphasizes the significance of this complication, which remains underdiagnosed despite its prevalence.

This topic has long been a concern in our practice. As early as 1994, we reported a case of fatal bilateral phrenic nerve injury following hypothermic open-heart surgery, underlining the potentially catastrophic impact of such injury, particularly when bilateral and unrecognized^[2]^. Our 2019 cohort study further evaluated diaphragmatic palsy in both adult and pediatric patients and showed that while some cases may resolve, others, especially in children, require active intervention such as diaphragm plication due to prolonged ventilatory dependency^[3]^. Contributing factors in adult include direct mechanical trauma during internal thoracic artery harvesting or pericardial dissection, as well as thermal injury from electrocautery or topical myocardial cooling. Excessive use of retractors, especially during off-pump procedures or redo sternotomies, may also lead to nerve stretching or compression.

In pediatric cardiac surgery, phrenic nerve palsy is more frequently associated with hypothermia-induced injury from the use of ice slush or cold saline during myocardial protection, especially in neonates and infants. Complex congenital procedures, such as the arterial switch or Fontan operations, involve extensive manipulation of cardiac structures near the phrenic nerve, increasing the risk of traction injury^[3,4]^.

Compared to the current study by Somers et al.^[1]^, which primarily focused on radiologic findings and spontaneous recovery, our clinical experience points to a wider spectrum of presentation, from benign to life-threatening. The variability in clinical outcome suggests a need for earlier identification and stratification of risk, especially in high-risk subgroups. We support the authors’ call for further research and additionally propose the incorporation of intraoperative nerve monitoring and early postoperative diaphragm imaging into standard protocols.

Recognition of this complication is critical not only for optimizing postoperative respiratory outcomes, but also for avoiding delays in extubation and unnecessary interventions.

## Artificial Intelligence Usage

The author declares use of ChatGPT to ease readability. The content produced by the artificial intelligence tool was revised and edited by the author as necessary, and he takes full responsibility for the content to be published.
